# Current status of the management of isolated syndesmotic injuries in Germany

**DOI:** 10.1007/s00402-022-04423-3

**Published:** 2022-04-11

**Authors:** Manuel Mutschler, Jan-Hendrik Naendrup, Thomas R. Pfeiffer, Vera Jaecker, Dariusch Arbab, Sven Shafizadeh, Tomas Buchhorn

**Affiliations:** 1 Witten/Herdecke University, Witten/Herdecke, Germany Alfred-Herrhausen-Straße 50, 58448; 2 Department of Foot Surgery, Waldkrankenhaus Bonn, Johanniter GmbH, Bonn, Germany Waldstraße 73, 53177; 3 Department of Trauma Surgery, Orthopaedic Surgery and Sports Traumatology, Witten/Herdecke University, Cologne Merheim Medical Centre, Cologne, Germany; 4 Department of Oncology, HaematologyInfectiology and Internistic Critical Care Medicine, University of Cologne, Cologne, Germany; 5 Department of Orthopaedic Surgery, Klinikum Dortmund, Dortmund, Germany; 6 Department of Trauma Surgery, Orthopaedic Surgery and Sports Traumatology, Sana Medical Centre Cologne, Cologne, Germany; 7 Foot and Ankle Department, Sporthopaedicum Straubing-Regensburg, Straubing, Germany

**Keywords:** Ankle syndesmotic injury, Syndesmotic screw fixation, Suture-button, MRI, Arthroscopy

## Abstract

**Introduction:**

Although non-fracture-related syndesmotic injuries of the ankle are relatively rare, they may lead to poor clinical outcome if initially undiagnosed or managed improperly. Despite a variety of literature regarding possibilities for treatment of isolated syndesmotic injuries, little is known about effective applications of different therapeutic methods in day-to-day work. The aim of this study was to assess the current status of the treatment of isolated syndesmotic injuries in Germany.

**Materials and methods:**

An online-questionnaire, capturing the routine diagnostic workup including clinical examination, radiologic assessment and treatment strategies, was sent to all members of the German Society of Orthopedic Surgery and Traumatology (DGOU) and Association of Arthroscopic and Joint Surgery (AGA). Statistical analysis was performed using Microsoft excel and SPSS.

**Results:**

Each question of the questionnaire was on average answered by 431 ± 113 respondents. External rotation stress test (66%), squeeze test (61%) and forced dorsiflexion test (40%) were most commonly used for the clinical examination. In the diagnostic workup, most clinicians relied on MRI (83%) and conventional X-ray analysis (anterior–posterior 58%, lateral 41%, mortise view 38%). Only 15% of the respondents stated that there is a role for arthroscopic evaluation for the assessment of isolated syndesmotic injuries. Most frequently used fixation techniques included syndesmotic screw fixation (80%, 42% one syndesmotic screw, 38% two syndesmotic screws), followed by suture-button devices in 13%. Syndesmotic screw fixation was mainly performed tricortically (78%). While 50% of the respondents stated that syndesmotic screw fixation and suture-button devices are equivalent in the treatment of isolated syndesmotic injuries with respect to clinical outcome, 36% answered that syndesmotic screw fixation is superior compared to suture-button devices.

**Conclusions:**

While arthroscopy and suture-button devices do not appear to be widely used, syndesmotic screw fixation after diagnostic work-up by MRI seems to be the common treatment algorithm for non-fracture-related syndesmotic injuries in Germany.

## Introduction

Injuries of the ankle are among the most common injuries in athletes and one of the most frequent musculoskeletal injuries presenting in the emergency department [1–3]. While sprains of the lateral ankle ligament complex constitute the most frequent type of ankle sprain and occur in about three-quarter of all ankle sprains, the incidence rate of acute syndesmotic ankle injuries is significantly lower at approximately 0.38 per 1000 athlete exposures, being defined as one athlete participating in one competition or practice [4]. In this context, male sex and higher level of competition are known risk factors for syndesmotic injuries [5]. Syndesmotic injuries mainly result from high-energy ankle trauma, especially involving external rotation force and excessive dorsiflexion, and occur isolated or in combination with other ligamentous or bony concomitant injuries [6, 7].

Although acute syndesmotic injuries are relatively rare, they can have debilitating long-term effects. If initially undiagnosed or managed improperly, they may lead to poor functional outcome, persisting ankle pain or post-traumatic osteoarthritis [8–11]. Therefore, early recognition and appropriate treatment seems to be pivotal. However, syndesmotic injuries are difficult to detect and distinguished from other ligamentous injuries of the ankle solely based on the history and physical examination [12]. Despite a variety of clinical tests, none of them entails a sufficient reliability and accuracy to identify a syndesmotic injury with adequate certainty [13]. Consequently, additional diagnostics such as X-ray, computed tomography (CT), magnet resonance imaging (MRI) or arthroscopy are used and recommended [14–17].

Regarding the treatment of syndesmotic injuries, several approaches are available, each with its own advantages, disadvantages and challenges: Arthroscopic versus open techniques, direct suture repairs and/or tibiofibular fixation methods including screws and suture-button devices [11, 14, 18–21]. Also regarding the appropriate and optimal postoperative care many protocols and controversies exist [22–24].

Beside the fact that many possibilities for the treatment of syndesmotic injuries exist, the current literature offers little information regarding the effective applications of different therapeutic methods in day-to-day clinical work. Due to this lack of descriptive statistics, a survey, consulting German clinicians, was implemented to gather the current status of the treatment of isolated syndesmotic injuries in Germany.

## Material and methods

Using a cloud-based survey software (https://www.surveymonkey.com/), an online-questionnaire about isolated syndesmotic injuries was designed and sent to all members of the German Society of Orthopaedics and Trauma (*Deutsche Gesellschaft für Orthopädie und Unfallchirurgie—DGOU*) as well as to the Society for Arthroscopy and Joint-Surgery (AGA—*Gesellschaft für Arthroskopie und Gelenkchirurgie*). One email reminder followed. The distribution of the questionnaire to the members was performed exclusively by the societies, therefore the total number of recipients as well as the response rate remains unknown due to an unknown number of respondents being member in both societies. The response to the questionnaire was collected anonymously, so that the content did not include direct hints about the participating clinics or physicians.

The survey contained 39 questions about the observed incidences of isolated syndesmotic injuries, the routine diagnostic workup with clinical examination and radiological assessment as well as current treatment strategies.

The data were statistically analyzed using Microsoft Excel and SPSS (version 26). As this investigation constituted a voluntary survey amongst medical professionals, no approval by the local ethics committee was required.

## Results

### Respondents characteristics

Each question of the online-questionnaire was on average answered by 431 ± 113 respondents, of which 76% (478/629) were trained as orthopedic and trauma surgeons, 36% (226/629) were general surgeons and 10% (66/629) were taking part in a residency program for trauma and orthopedic surgery. The vast majority of the respondents worked in hospitals (69%, 429/622), 19% (120/622) worked in private practice and 12% (73/622) stated that they work in a specialized department for foot and ankle surgery. In total, 18% (113/622) stated to be certified foot and ankle surgeons.

### Incidence of syndesmotic and associated injuries

The majority of respondents either treated one to ten patients (40% (237/593)) or up to 20 patients (35% (210/593)) with isolated syndesmotic injuries annually. Only 18% (101/593) stated to medicate 20 to 40 patients and 7% (41/593) more than 40 patients per year. In the respondent’s perspective (*n* = 404), associated injuries with acute syndesmotic lesions mainly involved the lateral ligament complex (51 ± 27% isolated anterior talofibular ligament (ATFL), 35 ± 23% combined ATFL/calcaneal fibular ligament (CFL) and 21 ± 22% ATFL/CFL/posterior talofibular ligament (PTFL) injuries). The deltoid ligament complex was seen affected in 31 ± 24% and posttraumatic bone edema as well osteochondral flake injuries were observed in 55 ± 27% and 21 ± 17%, respectively.

### Diagnostics in syndesmotic injuries

**Fig. 1:**
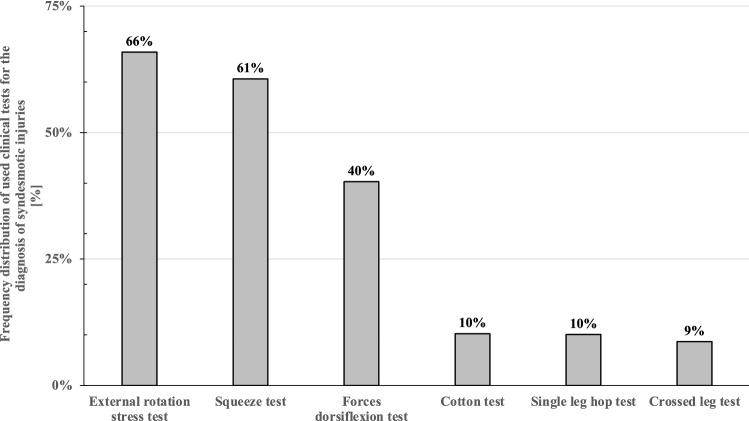
Frequency distribution of the clinical tests that respondents consider being meaningful for clinical decision making in the physical examination of syndesmotic injuries (multiple answers possible)

Asked about the meaning of the physical examination tests for clinical decision making, the majority of respondents (66%, 373/566) relied on the external rotation stress test as well as the squeeze test (61%, 343/566), followed by the forced dorsiflexion test (40%, 228/566). An overview of the physical examination tests used in day-to-day clinical work for isolated syndesmotic injuries is shown in Fig. 1.

**Fig. 2:**
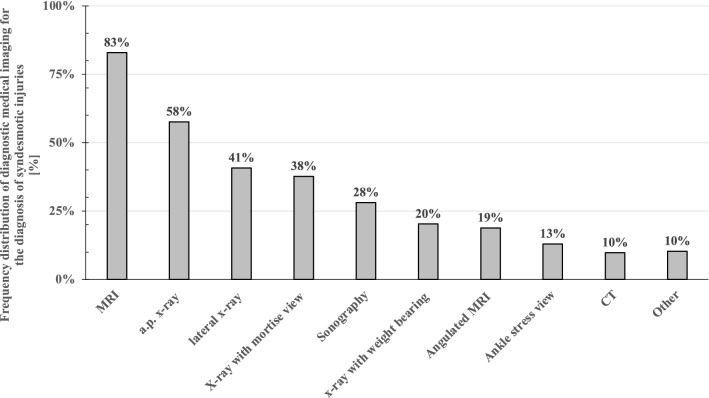
Frequency distribution of medical imaging that respondents consider being meaningful for clinical decision making for the diagnostic work-up of syndesmotic injuries (multiple answers possible)

As the respondents (n = 566) stated that the diagnosis of acute syndesmotic injury is missed in 49 ± 22% of the cases during the initial clinical examination on average, further diagnostics are needed. In the subsequent work-up, most clinicians relied on MRI (83%) and X-ray analysis (anterior–posterior X-ray (58%), lateral X-ray (41%), X-ray with mortise view (38%), anterior–posterior X-ray with weight bearing (20%)), as well as sonography (28%). Only 19% of the respondents preferred an angulated MRI parallel to the syndesmosis to confirm the diagnosis (n = 566, compare Fig. 2). 15% (86/562) of the respondents stated that there is a role for arthroscopic evaluation with a hook for the assessment of isolated syndesmotic injuries.

### Operative treatment strategies for acute syndesmotic injuries

According to the respondents (*n* = 593), 61 ± 35% of all isolated syndesmotic injuries were treated operatively in day-to-day clinical work. Indications for operative treatment were “unstable syndesmotic injuries” (97%) as well as injuries comprising all anatomic parts of the syndesmosis (93%). Isolated injuries of the anterior or posterior syndesmosis (25% and 24%, respectively) were less frequently considered as indication for surgery.

**Fig. 3:**
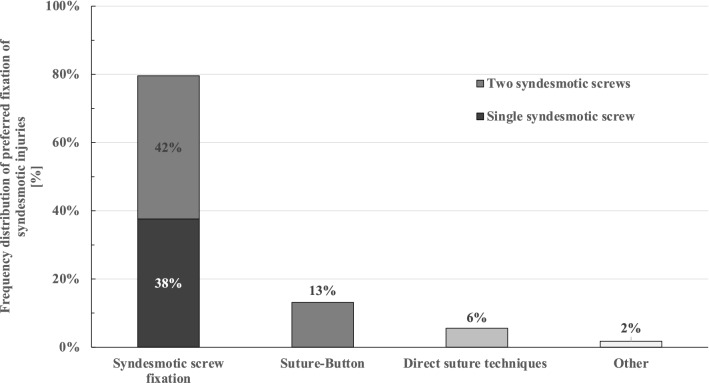
Frequency distribution of the preferred techniques for the operative stabilization of syndesmotic injuries

**Fig. 4:**
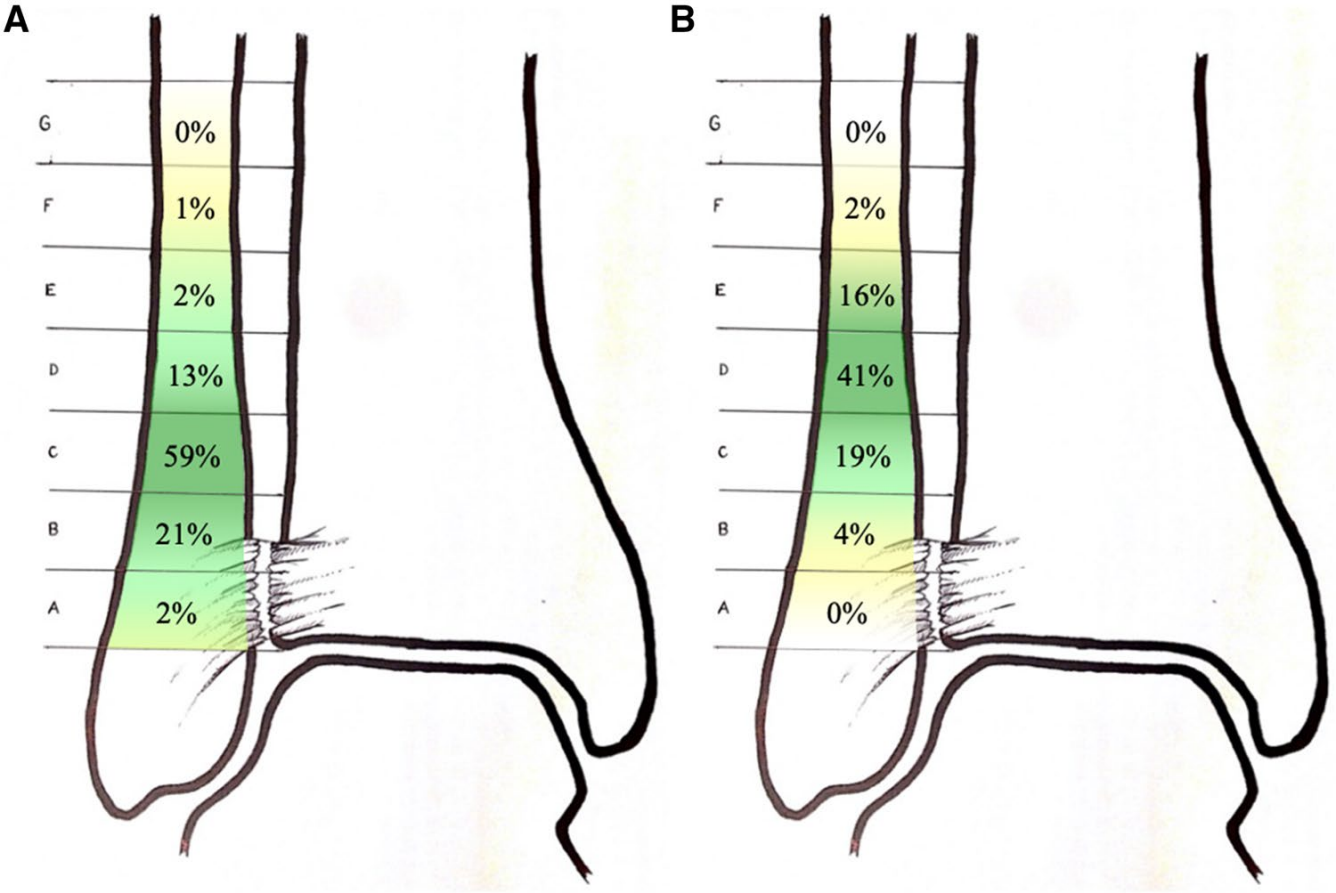
**A** Frequency distribution of the placement of the first syndesmotic screw. **B** Frequency distribution of the placement of the second syndesmotic screw if a second syndesmotic screw was placed (18% of the respondents do not place a second syndesmotic screw)

Different techniques for the operative stabilization of isolated syndesmotic injuries were used by the respondents in general: fixation with two syndesmotic screws (77%; 305/396), fixation with one syndesmotic screw (64%; 252/396), suture technique (44%; 175/396) and fixation with a suture-button device (41%; 162/396). However, when asked about the most frequently used technique, 80% of all respondents used syndesmotic screw fixation (42% one syndesmotic screw, 38% two syndesmotic screws), followed by suture-button devices in 13%, whereas direct suture techniques were used by 6% of the respondents (compare Fig. 3). Syndesmotic screw fixation was performed mainly tricortical (78%; 307/395), whereas quadricortical syndesmotic screw positioning was performed in 17% (67/395). The most frequently used positions for screws and suture-button devices are illustrated in Fig. 4.

Overall, 50% (199/395) of the respondents stated that syndesmotic screw fixation and suture-button devices are equivalent in the treatment of isolated syndesmotic injuries with respect to the clinical outcome, 36% (143/395) stated that screw fixation is superior to suture-button devices whereas 13% (53/395) found suture-button devices to be superior compared to syndesmotic screw fixation techniques.

Of all respondents, 97% (376/389) regularly perform an intraoperative assessment of the syndesmotic screw and suture-button positioning, hereby fluoroscopy (72%, 272/376) and 3D-fluroscopy (28%, 104/376) are the most commonly used technical devices. Intraoperative evaluation of the positioning of the fibula within the incisura was performed in 62% (243/389), mainly using fluoroscopy (59%, 144/243) and 3D-fluroscopy (47%, 115/243).

### Postoperative care

With respect to postoperative care, most syndesmotic screws were removed (92 ± 20%, *n* = 359), mainly after 6 weeks (92%), followed by 12 weeks (7%). Only 2% of the respondents stated not to remove the syndesmotic screws at all. In case of syndesmotic screw fixation, 96% (350/365) of the respondents stated not to allow full weight bearing until screws were removed. 70% (255/365) used an orthosis in the aftercare management, whereas walker devices (76%, 193/254) followed by ankle orthosis (30%, 75/254) and cast immobilization (16%, 41/254) were predominantly used. Also when using suture-button devices, most surgeons recommended partial weight bearing for 6 weeks (75%, 143/191). Orthoses were used in 82% (162/198), especially walker devices, (59%, 95/162), ankle orthoses (35%, 56/162) and cast immobilization (7%, 11/162).

## Discussion

Based on the Germany-wide online-survey, the presented results illustrate the current status of diagnostics and treatment of non-fracture-related syndesmotic injuries in Germany. The majority of surgeons rely on external rotation stress test and squeeze test for making the diagnosis of unstable isolated syndesmotic injuries. In contrast to arthroscopy, MRI is highly valued in the advanced diagnostic work-up of isolated syndesmotic injuries. Unstable and complete injuries of the syndesmosis are commonly treated with syndesmotic screw fixation (80%) with the vast majority of hardware being removed routinely in the postoperative follow-up. Suture-button devices continue to be less commonly used. However, it has to be stated that the results of the present survey only consist of pure descriptive statistics. Consequently, no therapeutic recommendations can be derived.

There is a variety of clinical tests to identify syndesmotic injuries, however, due to limited diagnostic accuracy clinicians cannot rely on a single test to diagnose the integrity of the syndesmosis with certainty [13]. This clinical problem is reflected in the present study, showing that the respondents use a variety of different tests, especially external rotation stress test, squeeze test and forced dorsiflexion test, to identify syndesmotic injuries. In accordance with the clinical usage of multiple diagnostic examinations, a recent meta-analysis proclaimed an initial clustering of test including high sensitivity tests (e.g., palpation or forced dorsiflexion), followed by clinical test with a high specificity (e.g., squeeze test) [25]. However, clinical tests alone are only of limited accuracy in the detection of isolated syndesmotic injuries and in the decision whether surgery is needed or not [13, 25]. This is in line with the results of the present study, where nearly half of the respondents indicated that clinical examination is not valid to thoroughly detect all fresh syndesmotic injuries and that MRI is frequently used in the advanced diagnostic work-up. However, MRI has only limited sensitivity for concomitant intra-articular injuries such as cartilage damage [26–28]. These lesions are reported to be associated with unstable injuries of the syndesmosis in up to 50% of patients [29]. The recent study also reported that at least 19% of these lesions needed therapeutic intervention, so consequently the current status of care is likely to overlook a potentially treatable concomitant injury. Therefore, arthroscopy was proposed for evaluation of both concomitant injuries and syndesmotic instability. In contrast to MRI examination, ankle arthroscopy allows direct visualization both statically as well as under applied stress load and high accuracy for the diagnosis of syndesmotic instabilities was demonstrated [17, 30–32]. Arthroscopy was therefore even proclaimed as the ultimate benchmark for the detection of syndesmotic instability due to its use in visualization of intraoperative joint reduction and evaluation of stability after fixation [33, 34]. Although arthroscopy is gaining importance in the treatment of ankle pathologies [35], in our study only 15% of the participants reported that arthroscopy is part of their diagnostic algorithm.

The treatment method of choice for injuries to all parts of the syndesmosis clearly appears to be surgical. However, only a quarter of the respondents indicated that they would consider surgical treatment for isolated injuries of the anterior or posterior syndesmosis, although cadaveric studies show that cutting the anterior syndesmosis already leads to significantly increased anterior–posterior translation and rotational instability of the fibula [36]. Knowing that even 1 mm of talus subluxation leads to significant change of contact forces in the tibiotalar joint, the risk of cartilage damage and osteoarthritis is potentially high even in isolated injuries of the AITFL (anterior inferior tibiofibular ligament) or PITFL (posterior inferior tibiofibular ligament) [37]. Thus, even in isolated injuries of the AITFL or PITFL, a thorough clinical examination should be performed and surgical therapy might be considered, especially in young and active patients.

In the last decade, suture-button devices have become increasingly popular and are extensively discussed in recent literature. Improved functional outcomes and rehabilitation as well as lower rates of broken implants are often cited as evidence to promote the usage of suture-button devices [38, 39]. Additionally, the data of the present study suggests that more than 90% of the respondents routinely remove the hardware after syndesmotic screw fixation, so consequently suture-button devices imply the advantage of being less invasive postoperatively. However, suture-button devices have not yet found its way into broad clinical application, as only 13% of the respondents reported using suture-button devices. While half of the respondents stated that syndesmotic screw fixation and suture-button devices are equivalent in the treatment of syndesmotic injuries, more than a third think that screw fixation is superior to suture-button devices. Therefore, the prolonged transfer into daily clinical work might be due to persistent doubt regarding the effectiveness of suture-button devices. However, a recent biomechanical analysis reinforced doubts, showing increased sagittal instability with ankle inversion after suture-button fixation while tricortical screw fixation restored the intact ankle tibiofibular kinematics [36]. In addition, the question, whether suture button devices are superior with respect to malreduction, remains controversial [40, 41]. Further biomechanical studies as well as clinical studies with long-term follow-up are needed to shine a light on this issue.

Evaluation of the correct alignment of the syndesmosis after reduction remains challenging. Intraoperative malrotations occur frequently and are associated with posttraumatic arthrosis as well as chronic syndesmotic instability. However, malrotations are difficult to detect especially when using conventional radiographs. That is why intraoperative 3D visualization is highly recommended [42, 43]. In contrast to this recommendation, only 28% of all respondents used 3D intraoperative visualization after syndesmotic fixation.

The present study also revealed that syndesmotic screws are routinely removed after 6 weeks. This contrasts with current literature mainly from the US, where a routine removal of syndesmotic screws is only advised in case of complaints related to hardware or malreduction and screws are left in situ for at least 3 to 9 months [43, 44]. Moreover, a recent systematic review illustrated no evidence to support routine removal of syndesmotic screws in regard to functional outcome, but found an association with higher financial costs and morbidity [45, 46]. Therefore, the current practice regarding routine hardware removal should be questioned critically.

The present study does have its limitations. As the entirety of recipients remained unclear, not every question addressed by all respondents and there was potential for multi-addressing, the basic population as well as the response rate cannot be reported. This entails a risk for selection bias that participating departments and clinicians do not mirror the current status of treatment of isolated syndesmotic injuries in Germany. High shares of clinicians treating less than ten non-fracture-related injuries of the syndesmosis underlines the rarity of this injury and entails the risk that no standard of care has been developed. Again, it has to be stated that the results of the present survey only consist of pure descriptive statistics and that no therapeutic recommendations can be derived. However, high shares of surgeons with small numbers of annual cases also indicate that probably a relatively wide cross section of clinicians was convinced to answer the questionnaire, which is indicative of the validity of the results.

## Conclusions

The treatment of non-fracture-related syndesmotic injuries is characterized by a variety of diagnostic and therapeutic possibilities. For the first time, a descriptive analysis was performed to describe this variety and to provide insight into the current status quo in Germany. Up to the present day, syndesmotic screw fixation after diagnostic work-up by MRI seems to be the most common treatment algorithm for non-fracture-related syndesmotic injuries. New fixation techniques, such as suture button devices, which have been shown to be at least equivalent with respect to clinical outcomes, do not appear to be in wide use in daily clinical routine. Most syndesmotic screws are removed after 6 weeks, while no evidence for an advantage of routine hardware removal exists. Although the clinical significance of arthroscopy and intraoperative 3D visualization has been scientifically shown, the present study illustrated that they seem to play only a minor role in daily clinical routine in Germany.

## References

[1] Fong D, Hong Y, Chan L (2007). A systematic review on ankle injury and ankle sprain in sports. Sports Med.

[2] Doherty C, Delahunt E, Caulfield B (2014). The incidence and prevalence of ankle sprain injury: a systematic review and meta-analysis of prospective epidemiological studies. Sports Med.

[3] Shah S, Thomas AC, Noone JM (2016). Incidence and cost of ankle sprains in United states emergency departments. Sports Health.

[4] Herzog MM, Kerr ZY, Marshall SW, Wikstrom EA (2019). Epidemiology of ankle sprains and chronic ankle instability. J Athl Train.

[5] Waterman B, Belmont P, Cameron K (2011). Risk factors for syndesmotic and medial ankle sprain: role of sex, sport, and level of competition. Am J Sports Med.

[6] Norkus SA, Floyd RT (2001). The anatomy and mechanisms of syndesmotic ankle sprains. J Athl Train.

[7] Lin C, Gross M, Weinhold P (2006). Ankle syndesmosis injuries: anatomy, biomechanics, mechanism of injury, and clinical guidelines for diagnosis and intervention. J Orthop Sports Phys Ther.

[8] DHooghe P, Joyce C, Hunt K, Kaux J (2018). Concomitant injuries in chronic ankle instability. Clin Res Foot Ankle.

[9] Choi WJ, Lee JW, Han SH (2008). Chronic lateral ankle instability. Am J Sports Med.

[10] Delco ML, Kennedy JG, Bonassar LJ, Fortier LA (2017). Post-traumatic osteoarthritis of the ankle: a distinct clinical entity requiring new research approaches. J Orthop Res.

[11] Gribble P, Bleakley C, Caulfield B (2016). Evidence review for the 2016 International ankle consortium consensus statement on the prevalence, impact and long-term consequences of lateral ankle sprains. Br J Sports Med.

[12] Mulligan E (2011). Evaluation and management of ankle syndesmosis injuries. Phys Ther Sport.

[13] Sman ADA, Hiller CEC, Refshauge KKM (2013). Diagnostic accuracy of clinical tests for diagnosis of ankle syndesmosis injury: a systematic review. Br J Sports Med.

[14] Hunt KJ (2013). Syndesmosis injuries. Curr Rev Musculoskelet Med.

[15] Oae K, Takao M, Naito K (2003). Injury of the tibiofibular syndesmosis: value of MR imaging for diagnosis. Radiology.

[16] Takao M, Ochi M, Oae K (2003). Diagnosis of a tear of the tibiofibular syndesmosis. The role of arthroscopy of the ankle. J Bone Joint Surg Br.

[17] Krähenbühl N, Weinberg N, Davidson N (2018). Imaging in syndesmotic injury: a systematic literature review. Skeletal Radiol.

[18] Rammelt S, Obruba P (2015). An update on the evaluation and treatment of syndesmotic injuries. Eur J Trauma Emerg Surg.

[19] Akoh C, Phisitkul P (2019). Anatomic ligament repairs of syndesmotic injuries. Orthop Clin North Am.

[20] Liu G, Chen L, Gong M (2019). Clinical evidence for treatment of distal tibiofibular syndesmosis injury: a systematic review of clinical studies. J Foot Ankle Surg.

[21] Zhang P, Liang Y, He J (2017). A systematic review of suture-button versus syndesmotic screw in the treatment of distal tibiofibular syndesmosis injury. BMC Musculoskelet Disord.

[22] Williams GN, Allen EJ (2010). Rehabilitation of syndesmotic (high) ankle sprains. Sports Health.

[23] Latham AJ, Goodwin PC, Stirling B, Budgen A (2017). Ankle syndesmosis repair and rehabilitation in professional rugby league players: a case series report. BMJ Open Sport Exerc Med.

[24] Brosky T, Nyland J, Nitz A, Caborn D (1995). The ankle ligaments: consideration of syndesmotic injury and implications for rehabilitation. J Orthop Sports Phys Ther.

[25] Netterström-Wedin F, Bleakley C (2021). Diagnostic accuracy of clinical tests assessing ligamentous injury of the ankle syndesmosis: a systematic review with meta-analysis. Phys Ther Sport.

[26] O’Neill PJ, Van Aman SE, Guyton GP (2010). Is MRI adequate to detect lesions in patients with ankle instability?. Clin Orthop Relat Res.

[27] Gatlin CC, Matheny LM, Ho CP (2015). Diagnostic accuracy of 3.0 tesla magnetic resonance imaging for the detection of articular cartilage lesions of the talus. Foot Ankle Int.

[28] Bauer JS, Barr C, Henning TD (2008). Magnetic resonance imaging of the ankle at 3.0 tesla and 1.5 tesla in human cadaver specimens with artificially created lesions of cartilage and ligaments. Invest Radiol.

[29] Rellensmann K, Behzadi C, Usseglio J (2020). Acute, isolated and unstable syndesmotic injuries are frequently associated with intra-articular pathologies. Knee Surg Sport Traumatol Arthrosc.

[30] Lucas DE, Watson BC, Simpson GA (2016). Arthroscopic evaluation of syndesmotic instability and malreduction. Foot Ankle Spec.

[31] Hagemeijer NC, Elghazy MA, Waryasz G (2021). Arthroscopic coronal plane syndesmotic instability has been over-diagnosed. Knee Surg Sports Traumatol Arthrosc.

[32] Turky M, Menon KV, Saeed K (2018). Arthroscopic grading of injuries of the inferior tibiofibular syndesmosis. J Foot Ankle Surg.

[33] Kellett JJ, Lovell GA, Eriksen DA, Sampson MJ (2018). Diagnostic imaging of ankle syndesmosis injuries: a general review. J Med Imaging Radiat Oncol.

[34] Nishikawa DRC, Saito GH, de Oliveira Junior AS (2021). Clinical outcomes of isolated acute instability of the syndesmosis treated with arthroscopy and percutaneous suture-button fixation. Arch Orthop Trauma Surg.

[35] Vega J, Karlsson J, Kerkhoffs GMMJ, Dalmau-Pastor M (2020). Ankle arthroscopy: the wave that’s coming. Knee Surg Sport Traumatol Arthrosc.

[36] Patel NK, Murphy CI, Pfeiffer TR (2020). Sagittal instability with inversion is important to evaluate after syndesmosis injury and repair: a cadaveric robotic study. J Exp Orthop.

[37] Ramsey PL, Hamilton W (1976). Changes in tibiotalar area of contact caused by lateral talar shift. J Bone Jt Surg Ser A.

[38] Shimozono Y, Hurley ET, Myerson CL (2019). Suture button versus syndesmotic screw for syndesmosis injuries: a meta-analysis of randomized controlled trials. Am J Sports Med.

[39] Andersen MR, Frihagen F, Hellund JC (2018). Randomized trial comparing suture button with single syndesmotic screw for syndesmosis injury. J Bone Joint Surg Am.

[40] Sanders D, Schneider P, Taylor M (2019). Improved reduction of the tibiofibular syndesmosis with tightrope compared with screw fixation: results of a randomized controlled study. J Orthop Trauma.

[41] Hennings R, Spiegl UJ, Fuchs C (2021). Does the orientation of syndesmosis fixative device affect the immediate reduction of the distal tibiofibular joint?. Arch Orthop Trauma Surg.

[42] Franke J, von Recum J, Suda AJ (2012). Intraoperative three-dimensional imaging in the treatment of acute unstable syndesmotic injuries. J Bone Joint Surg Am.

[43] Rammelt S, Manke E (2018). Syndesmosenverletzungen. Unfallchirurg.

[44] Walley KC, Hofmann KJ, Velasco BT, Kwon JY (2017). Removal of hardware after syndesmotic screw fixation: a systematic literature review. Foot Ankle Spec.

[45] Dingemans SA, Rammelt S, White TO (2016). Should syndesmotic screws be removed after surgical fixation of unstable ankle fractures? A systematic review. Bone Joint J.

[46] Lalli TAJ, Matthews LJ, Hanselman AE (2015). Economic impact of syndesmosis hardware removal. Foot (Edinb).

